# Ongoing measles outbreaks in Romania, March 2023 to August 2023

**DOI:** 10.2807/1560-7917.ES.2023.28.35.2300423

**Published:** 2023-08-31

**Authors:** Mihaela Lazar, Cătălina Pascu, Maria Roșca, Aurora Stănescu

**Affiliations:** 1 Cantacuzino National Military Medical Institute for Research and Development, Bucharest, Romania; 2 National Centre for Communicable Diseases Surveillance and Control, National Institute of Public Health, Bucharest, Romania

**Keywords:** measles, MMR, immunisation, outbreak, Romania

## Abstract

Measles cases have recently been increasing in Romania, with 460 confirmed cases by August 2023. From March 2023, measles cases increased, and outbreaks were recorded in Cluj, Bistrita Nasaud, Sibiu and Mures counties. New D8 virus variants were identified, different from the variants detected in Romania before the COVID-19 pandemic. We here provide epidemiological and molecular data on the current increase in measles cases in the context of the measles elimination process in the World Health Organization European Region.

Here we report an ongoing measles outbreak in Romania. Following a 2-year period with zero indigenous measles cases, the disease reappeared in late 2022. Outbreaks of measles occurred in March 2023 in the northern part of Romania and by August 2023, 460 of 580 possible measles cases were confirmed in 17 of the 41 Romanian counties. Most cases were registered among unvaccinated children. No measles-related deaths have so far been notified. 

## Case definition and notification of measles in Romania

Case-based surveillance of measles has been conducted continuously since 2005 based on the European Union case definition [1]. A possible case was a person with fever, maculopapular rash and at least one of the following symptoms: cough, coryza, or conjunctivitis. A confirmed case was a case not recently vaccinated and meeting the clinical definition and the following laboratory criteria: measles IgM antibody in serum samples or isolation of measles virus (MV) from a clinical specimen or detection of MV-specific nucleic acid from a clinical specimen using PCR or IgG seroconversion or a significant rise in measles IgG antibody (validated method). 

Notification of a patient with measles has been compulsory by law in Romania since 1978 and medical practitioners have to immediately report all cases with fever and maculopapular rash and at least one of the following symptoms: cough, coryza or conjunctivitis (possible measles cases) to the local public health authorities. Blood samples and/or swabs are collected from possible cases and sent to sub-national laboratories and the national laboratory for measles laboratory confirmation for case investigation and control measures, including contact tracing. At national level, the National Centre for Communicable Diseases Surveillance and Control in Bucharest collects and analyses all notifications of measles cases [2]. Following the introduction of measles vaccination in Romania in 1979, epidemics of measles have occurred every 2–4 years: in 1982, 1986, 1993, 1997, 2003, 2010 and 2016 [3,4].

## Measles cases in the current outbreak in Romania

Measles virus-specific IgM antibodies were detected with commercial enzyme-linked immunosorbent assays kits (ELISA Euroimmum - native antigens and recombinant nucleoprotein) primarily used in the laboratory network for laboratory confirmation.

**Figure 1:**
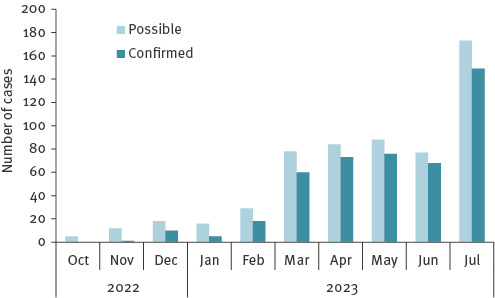
Possible and confirmed measles cases by month, Romania, October 2022–July 2023 (n = 580)

**Figure 2:**
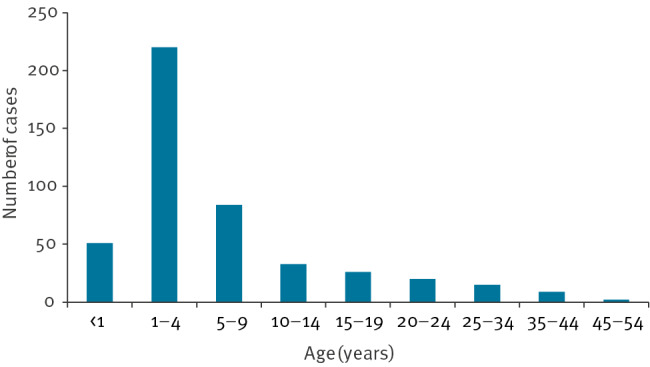
Age distribution of confirmed measles cases by month, Romania, October 2022–July 2023 (n = 460)

Since December 2022, measles activity has been increased, with ongoing outbreaks in several counties in Romania. The primary case could not be identified, and the first confirmed cases had no history of travel abroad. From October 2022 to the end of July 2023, 460 of 580 possible measles cases were confirmed (Figure 1). The median age of the confirmed cases was 4 years (range: 42 days–48 years), 237 (51.5%) were female and 223 (48.5%) were male (Figure 2). Of the 460 confirmed measles cases, 362 (78%) were among unvaccinated individuals, 51 were too young to be eligible for vaccination, 15 had incomplete vaccination (one dose of MMR), and for the remaining 32, this information not available. 

## Genotype distribution

**Figure 3:**
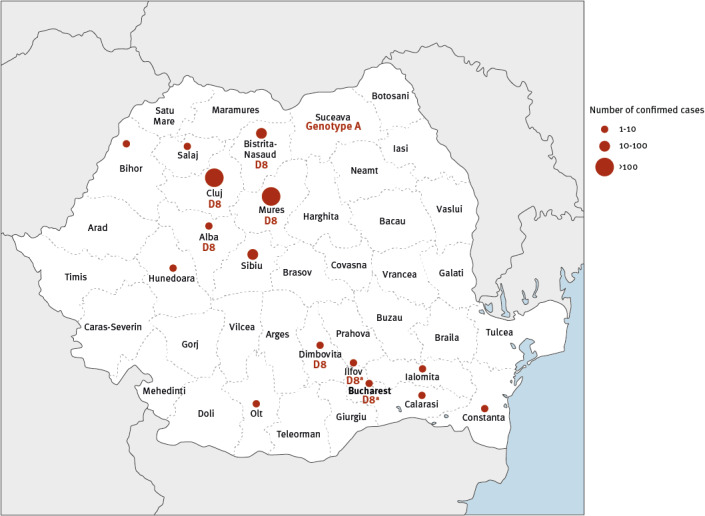
Geographical distribution of confirmed measles cases, Romania, October 2022–July 2023 (n = 460)

^a^ Two different D8 clusters were detected in this county.

Throat swabs were collected from 228 patients with clinically diagnosed measles and sent to the National Laboratory for Measles and Rubella at Cantacuzino National Military-Medical Institute for Research and Development, Bucharest for confirmation by real time reverse transcription PCR (RT-PCR). Positive samples with quantification cycle < 30 (n = 61) were selected for MV genotyping. In order to determine the MV genotype, we obtained the sequence of the 450 nt (N-450) coding for the carboxyl-terminal 150 amino acids of the MV nucleoprotein (N), according with CDCs protocol [5] from 61 positive samples collected from cases in eight counties. Except one sequence clustered within clade A (includes all vaccine strains of measles) identified in Suceava county, all sequences belonged to genotype D8 (Figure 3).

Genotypes detected in selected samples in the counties are indicated in red.

Wild-type MV are divided in eight clades designated A to H containing 24 genotypes based on the nucleotide sequences of their haemagglutinin (H) and N genes, which are the most variable genes in the viral genome. For each genotype, a reference strain is designated for phylogenetic analysis, usually the earliest known virus isolate of that group. Within a genotype there may be multiple distinct genetic lineages [6].

## Phylogenetic analysis

**Figure 4:**
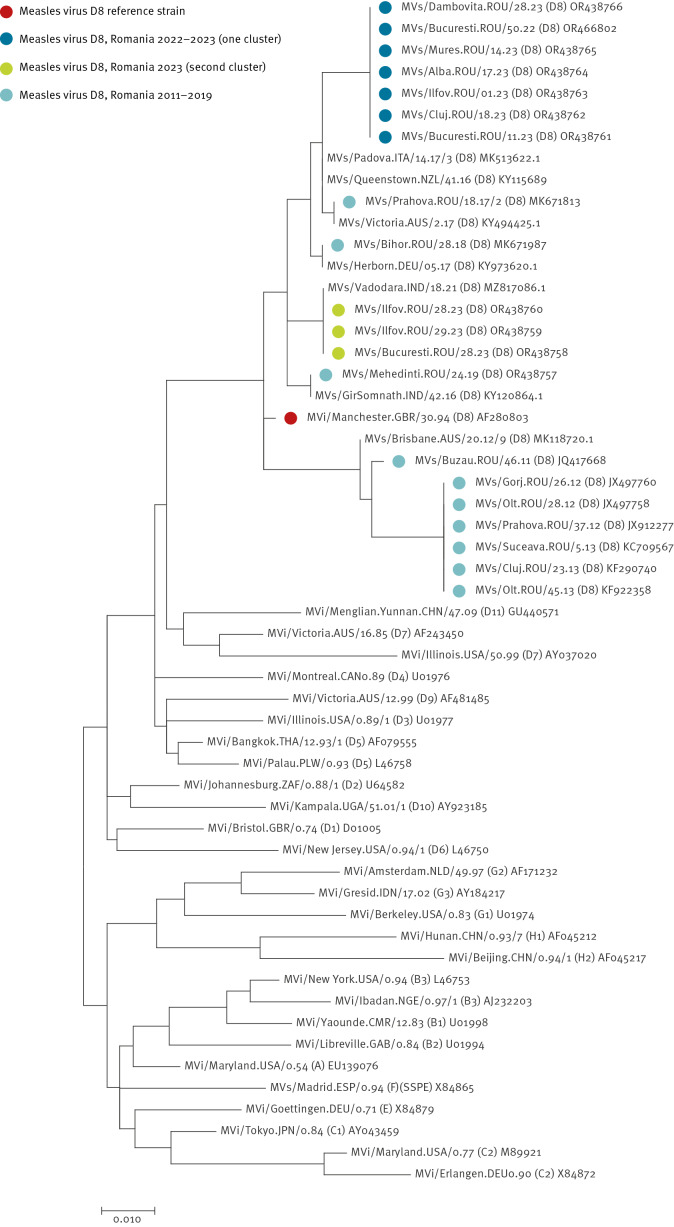
Phylogenetic relationship between representative measles strains of genotype D8, Romania, 2023 outbreak, compared with historical sequences (n = 20)

ROU: Romania; WHO: World Health Organization.

We performed a maximum-likelihood phylogenetic analysis constructed by MEGA–X [7] with 20 representative full-length MV sequences isolated in 2011–2023 (one example of identical sequences per county; GenBank numbers: OR438757- OR438766, OR466802, JX497760, JX497758, JX912277, KC709567, KF290740, KF922358, MK671813, MK671987 and JQ417668), the genotype reference strain from the WHO’s list for genotyping, and non-Romanian genotype D8 sequences from the same period available in GenBank and the Measles Virus Nucleotide Surveillance (MeaNS2) database [8-10]. We identified two D8 clusters in the 2023 outbreak in Romania (Figure 4). In the first D8 cluster, a family with four members with a history of travel in Thailand was confirmed with measles in December, MVs/Bucuresti.ROU/50.22 OR466802. This MV sequence was identical to sequences from not epidemiologically linked cases from Cluj, Alba, Mures, Dambovita, Bucharest and Ilfov counties from January to July and to sequences from Australia, Indonesia and Malaysia in the MeaNS2 database (data not shown). Sequences collected from Bucharest and Ilfov County formed a second D8 cluster and were identical with sequences from India, MVs/Vadodara.IND/18.21 MZ817086.1. All sequences in our current outbreak were different from D8 sequences collected from the Romanian measles outbreaks from 2011 to 2019, suggesting multiple introductions of MV.

The evolutionary history was inferred by using the maximum likelihood method and Tamura–Nei model by applying neighbour-join and BioNJ algorithms to a matrix of pairwise distances estimated using the maximum composite likelihood approach and then selecting the topology with superior log likelihood value. Evolutionary analyses were conducted in MEGA X and edited with Inskape.

## Discussion

Romania has recently experienced a resurgence of measles after the COVID-19 pandemic. Measles is a highly infectious disease that can result in serious complications such as pneumonia, visual impairment, severe diarrhoea and related dehydration, ear infections or encephalitis. There is no specific antiviral therapy for the treatment of measles, but the disease can be prevented by vaccination. 

Although safe and cost-effective vaccines exist, the World Health Organization (WHO) estimated 9 million measles cases with 128,000 deaths worldwide in 2021, affecting young children in particular [11]. In the early months of 2022, the number of measles cases was ca 79% higher than in the same period in 2021, and experts highlighted an increased risk of measles outbreaks in 2023 [12]. In 2023, 17 countries in the WHO European Region had reported measles cases by April 2023, and the number of cases in the first 2 months exceeded the annual total of 2022 [13].

The WHO recommends maintaining vaccination coverage of at least 95% in order to achieve the target of measles elimination [14]. According to the data provided by the National Centre for Surveillance and Control of Communicable Diseases, immunisation coverage with the first dose of measles–mumps–rubella (MMR) in Romania was 62% in 2022 and increased to 78% in 2023 for children born in 2021 [15]. One of the reasons for the low vaccination coverage in Romania could be parents who chose not to vaccinate their children. Another explanation for the low vaccination rates during the COVID 19 pandemic could be decreased access to general practitioners following movement restrictions and by the concentration of human resources from the health system in the pandemic management [16-18]. Control measures were implemented to improve uptake of MMR vaccine, for example offering catch-up vaccination to children that had not followed the recommended dosing schedule and lowering the vaccination age from 1 year to 9–11 months.

The low measles case numbers reported in 2020 in Romania could be the effect of restrictions during the COVID-19 pandemic. Here we applied molecular surveillance to investigate the development of the measles situation in Romania in order to provide insights in the circulation of MV and understand whether there is endemic transmission or multiple MV introductions. The missing identification of the primary case is problematic as it prevented early contact tracing and follow-up. Until measles is eliminated in the WHO European Region, vigilance is required to identify cases with symptoms of measles and minimise the risk of secondary cases.

## Conclusions

A measles outbreak with genotype D8 is ongoing in Romania. Insufficient MMR coverage has led to an accumulation of susceptible individuals. Measles transmission was difficult to assess through epidemiological information alone because links between the cases could not be identified. The molecular epidemiology suggests that there are different chains of transmission, indicating active transmission of measles in the country.
